# Long-Term Breast Cancer Risk in Hodgkin Lymphoma Survivors: Evaluating Background Parenchymal Enhancement and Radiotherapy-Induced Toxicity

**DOI:** 10.3390/cancers16234091

**Published:** 2024-12-06

**Authors:** Filomena Emanuela Laddaga, Michele Telegrafo, Carmela Garzillo, Alba Fiorentino, Angela Sardaro, Stefano Martinotti, Marco Moschetta, Francesco Gaudio

**Affiliations:** 1 Hematology and Cell Therapy Unit, IRCCS Istituto Tumori “Giovanni Paolo II”, 70124 Bari, Italy; 2 Interdisciplinary Department of Medicine (DIM), Section of Radiology and Radiation Oncology, University of Bari, 70124 Bari, Italy; 3 Department of Medicine and Surgery, LUM University “Giuseppe Degennaro”, Casamassima, 70010 Bari, Italymartinotti@lum.it (S.M.)

**Keywords:** Hodgkin, long term survivors, late toxicity, radiotherapy, breast cancer

## Abstract

Hodgkin lymphoma (HL) treatment has dramatically improved, with high survival rates in early stages. However, long-term survivors face an increased risk of secondary cancers, particularly breast cancer (BC), which emerge as a leading cause of mortality decades after therapy. **Background/Objectives**: This study explores the risk of BC and the toxic effects of radiation therapy (RT) in long-term HL survivors compared to age-matched high-risk women, including BRCA1 and BRCA2 mutation carriers. A prospective study was conducted on 62 women who had undergone chemotherapy and involved-field RT for HL, with MRI used to assess breast tissue changes. This study’s primary endpoint was to analyze BC incidence in HL survivors, while secondary objectives focused on the analysis of background parenchymal enhancement (BPE) in irradiated areas. **Results**: The findings revealed a 5% incidence of BC in HL survivors, with 50% showing moderate or marked BPE, similar to that observed in high-risk BC controls. No significant differences in BPE distribution were found between the two groups. **Conclusions**: The study highlights the long-term risk of BC in HL survivors and suggests that advanced RT techniques and targeted therapies may help reduce the incidence of secondary tumors. Future research should focus on understanding the genetic and biological mechanisms behind treatment-induced cancers

## Simple Summary

This study looks at the risk of developing breast cancer in survivors of Hodgkin lymphoma (HL), especially those who received chest radiation as part of their treatment. HL survivors are at a higher risk of breast cancer, and this risk increases over time. The research shows that radiation therapy used to treat HL can cause long-term damage to breast tissue, leading to an increased chance of developing cancer later on. The study also found that early screening and continuous monitoring are important for detecting cancer early in these survivors. Overall, it highlights the need for special care and surveillance for people who survived Hodgkin lymphoma, especially if they had been subjected to chest radiation.

## 1. Introduction

Currently, Hodgkin lymphoma (HL) has an excellent clinical outcome, with an overall survival rate of approximately 90% in early-stage cases [1,2]. However, 15–30 years after therapy, cumulative mortality due to second cancers exceeds deaths resulting from HL. Among these secondary cancers, breast cancer (BC) represents the highest absolute risk for HL survivors [3,4,5,6].

It is widely accepted that HL is highly sensitive to radiotherapy (RT). In early-stage HL, involved-field RT (IF-RT) with 20–30 Gray (Gy), combined with first-line chemotherapy consisting of adriamycin, bleomycin, vinblastine, and dacarbazine (ABVD regimen), remains the gold standard for disease treatment. Given the young age of most patients at diagnosis and their long survival, increasing attention has been focused on the long-term toxicity of treatments. RT can induce cardiovascular disease, compromise thyroid and lung function, and, most notably, lead to the development of secondary malignancies [2,7,8].

Secondary malignancies are the leading cause of death in patients with a history of HL, with breast cancer conferring an eightfold increased risk [3,9,10].

Recently, it has been hypothesized that magnetic resonance imaging (MRI) characteristics of normal breast tissue may provide more precise information about BC risk than traditional mammographic density. Despite the increased risk of breast cancer in Hodgkin lymphoma survivors, limited studies have directly compared MRI characteristics, such as background parenchymal enhancement (BPE), between these patients and high-risk individuals, such as BRCA mutation carriers. This gap in the literature underscores the need for further investigation into the role of MRI in assessing breast cancer risk in HL survivors, particularly in light of the long-term effects of radiotherapy.

BPE, resulting from dynamic contrast-enhanced MRI, has recently been shown to correlate with breast cancer risk. It refers to the increase in normal-appearing fibroglandular tissue, which is evaluated on contrast-enhanced images at the first time point after contrast-medium injection. BPE reflects the vascularity of the breast parenchymal tissue and varies with hormonal changes. It changes with the menstrual cycle, increasing with hormone replacement therapy, and decreasing with bilateral salpingo-oophorectomy, menopause, and anti-estrogenic therapies [11,12,13,14,15,16,17,18].

The BRCA1 and BRCA2 genes are involved in DNA repair and cell cycle checkpoint control and are considered tumor suppressor genes. The risk of developing BC is approximately 85% in BRCA1 mutation carriers and approximately 45% in BRCA2 mutation carriers. Therefore, the American Cancer Society recommends annual breast MRI screening starting at age 30 for all BRCA mutation carriers [12,19,20].

We hypothesize that MRI-based background parenchymal enhancement (BPE) measurements could be a reliable predictor of breast cancer risk in HL survivors, similar to the risk observed in BRCA mutation carriers. Additionally, we aim to investigate whether radiotherapy exposure influences BPE patterns and contributes to the development of breast cancer in this patient group. This prospective study evaluates the toxic effects of RT and the risk of developing BC in long-surviving HL patients, comparing them to age-matched women who underwent breast-screening MRI for high-risk groups (BRCA1 and BRCA2 carriers). The results of this study may provide valuable insights into more personalized breast cancer screening strategies for HL survivors, potentially leading to earlier detection and improved outcomes for this high-risk population. By identifying MRI-based biomarkers for breast cancer risk, clinicians may be better equipped to manage the long-term care of HL survivors.

## 2. Materials and Methods

This prospective study focused on 62 women, aged 22–49 years (median age 35 years), with a history of prior early Hodgkin lymphoma, treated with chemotherapy and in-field radiation therapy for at least 15 years. MRIs were compared against 62 age-matched women with no prior history of cancer who underwent annual risk-based breast cancer surveillance for high-risk individuals (BRCA1 or BRCA2 mutation carriers).

Written informed consent was obtained from all patients according to the principles of the Declaration of Helsinki.

### 2.1. Study Endpoint

The main objectives of the study are as follows:

- To analyze the incidence of breast cancer in patients treated with chemotherapy and chest radiation for Hodgkin lymphoma;
- To compare the risk of breast cancer in Hodgkin lymphoma survivors with age-matched women at high risk for breast cancer, such as those with BRCA1 or BRCA2 mutations;
- To examine and compare the background parenchymal enhancement (BPE) in irradiated areas of patients with Hodgkin lymphoma versus those without a history of cancer.

The secondary objective is to analyze the distribution of BPE in Hodgkin lymphoma survivors and women without cancer history, in order to better understand its role as a risk indicator for developing breast cancer.

### 2.2. Study Parameters and Variables

The study considered a variety of parameters and variables to assess the breast cancer risk in long-term Hodgkin lymphoma survivors, focusing on the effects of radiotherapy and chemotherapy. These include the following:

#### 2.2.1. Patient Demographics

- Age: the age range of the study participants was between 22 and 49 years, with a median age of 35 years at the time of MRI examination;
- Age at Treatment: participants were treated for Hodgkin lymphoma at a median age of 25 years (range 12–36 years);
- Time Since Treatment: the median time since receiving radiotherapy was 15 years (range 10–19 years).

#### 2.2.2. Radiotherapy Parameters

- Type of Radiotherapy: Involved-field radiotherapy (IF-RT) was used, delivering 30 Gy in 15 fractions (2 Gy per fraction). This focused on the thoracic region, including lymph node areas affected by Hodgkin lymphoma;
- Radiation Dose: a total dose of 30 Gy was administered using 3D conformal radiotherapy (3DCRT) techniques.

#### 2.2.3. Chemotherapy

- Chemotherapy Regimen: all patients received the ABVD regimen (adriamycin, bleomycin, vinblastine, and dacarbazine) with four cycles, each lasting 28 days, administered on days 1 and 15 of each cycle.

#### 2.2.4. Breast Cancer Risk Assessment:

- Background Parenchymal Enhancement (BPE): The degree of normal breast tissue enhancement after gadolinium contrast administration was measured using MRI. BPE was classified into the following four categories: minimal (Type 1), mild (Type 2), moderate (Type 3), and marked (Type 4);
- Breast MRI Findings: MRI was used to evaluate breast tissue characteristics, including BPE and the presence of any abnormal lesions or malignant findings. The MRI sequences included T1-weighted imaging, T2-weighted imaging, dynamic contrast-enhanced imaging, and subtraction imaging for detecting enhancing lesions.

#### 2.2.5. Control Group Variables

- High-Risk Breast Cancer Cohort: a comparison group of 62 age-matched women at high risk of breast cancer, such as those with BRCA1 or BRCA2 mutations, was used for comparison;
- BPE Distribution in Control Group: BPE patterns were also evaluated in the control group to compare breast tissue characteristics between Hodgkin lymphoma survivors and high-risk women without prior cancer.

#### 2.2.6. Malignant Lesions Detection

Incidence of Breast Cancer: The primary endpoint was the incidence of breast cancer among the study participants. Any detected breast cancers were categorized and related to BPE type.

These variables and parameters were systematically analyzed to investigate the potential risk factors for breast cancer in Hodgkin lymphoma survivors, particularly focusing on the effects of radiotherapy and the role of BPE as an indicator of breast cancer risk.

### 2.3. Magnetic Resonance Imaging Protocol

MRI examinations were performed on a 1.5 T MRI device (Achieva, Philips Medical Systems, Best, The Netherlands) by using a 16-channel breast coil. The following protocol was used: transverse short TI inversion recovery (STIR) turbo-spin-echo (TSE) sequence (TR/TE/TI = 3.800/60/165 ms, field of view (FOV) = 250 × 450 mm^2^ (AP × RL), matrix 168 × 300, 50 slices, 3 mm slice thickness without gaps, 3 averages, and turbo factor 23, resulting in a voxel size of 1.5 × 1.5 × 3.0 mm^3^); transverse T2-weighted TSE (TR/TE = 6.300/130 ms, FOV = 250 × 450 mm^2^ (AP × RL), matrix 336 × 600, 50 slices, 3 mm slice thickness without gaps, 3 averages, turbo factor 59, and SENSE factor 1.7, resulting in a voxel size of 0.75 × 0.75 × 3.0 mm^3^); and three-dimensional (3D) dynamic, contrast-enhanced (CE) T1-weighted high resolution isotropic volume (THRIVE) sequences (TR/TE = 4.4/2.0 ms, FOV = 250 × 450 × 150 mm^3^ (AP × RL × FH), matrix 168 × 300, 100 slices, 1.5 mm slice thickness, turbo factor 50, SENSE factor 1.6, 6 dynamic acquisitions, 1.5 mm^3^ isotropic voxels, a dynamic data acquisition time of 1 min 30 s, and a total sequence duration of 9 min). Gadobutrol (Gadovist, Bayer, Berlin, Germany) was intravenously injected at a dose of 0.1 mmol/kg of body weight and a flow rate of 1.5 mL/s, followed by 20 mL of saline solution. Image subtraction sequences were created in order to diagnose all enhancing lesions.

### 2.4. Image Analysis

All MRI data were analyzed on a diagnostic workstation equipped with dedicated software for MRI examination (View-ForumR5.1 V1L1 2006). Two radiologists with more than 5 years of experience in the field of breast MRI, blinded to the patient history and to clinical, DBT, and ultrasound findings, evaluated all subtracted and 3D maximum intensity projection (MIP) MR-enhanced images for classifying normal BPE.

In the case of disagreement between the two radiologists on the classification of BPE or MRI interpretation, a consensus approach was used. The radiologists reviewed the images together and discussed their findings to reach a mutual agreement. If consensus could not be reached, a third experienced radiologist was consulted to provide a final interpretation. This process ensured the reliability and accuracy of the results.

BPE was classified into four categories: minimal or Type 1 (<25% of glandular tissue enhancement), mild or Type 2 (25–50% enhancement), moderate or Type 3 (50–75% enhancement), and marked or Type 4 (>75%). If MRI lesions were suspected, BPE was assessed on the contralateral breast to avoid any confounding effects. The post-processing mean duration time was approximately 10 min for MR post-contrast imaging.

### 2.5. Statistical Analysis

Patient characteristics were assessed with summary statistics for the two groups. A *t*-test for independent samples was used to compare differences in age and BPE type distribution between the two patient groups. The distribution of BPE patterns within the two groups of patients was calculated, and the χ^2^ test was used to evaluate the significance of BPE type distribution in each group. A multivariate regression analysis, including patient age at the time of breast MRI, age at the time of radiotherapy, and years since radiotherapy, was used to assess any confounding effects on BPE measurement and distribution among the case group.

Cohen’s kappa statistics were used to assess inter-observer agreement for classifying BPE. A k-value of more than 0.81 was considered an almost perfect agreement, with values of 0.61–0.80 and 0.41–0.60 considered substantial and moderate agreements, respectively.

All calculations were performed using the NCSS2007^®^ (Version 7.0) statistical software.

## 3. Results

A total of 62 women with a history of Hodgkin lymphoma (HL) who had undergone chemotherapy and in-field radiation therapy for at least 15 years were included in the study. The control group consisted of 62 age-matched women with no prior history of cancer, selected for high-risk breast cancer surveillance due to BRCA1 or BRCA2 mutations.

The median age of HL survivors at the time of MRI examination was 35 years (range: 22–49 years). The median age at the time of Hodgkin lymphoma treatment was 25 years (range: 12–36 years), with a median of 15 years (range: 10–19 years) since the completion of radiotherapy.

The control group had similar age characteristics, with a median age of 34 years (range: 23–49 years).

**Figure 1:**
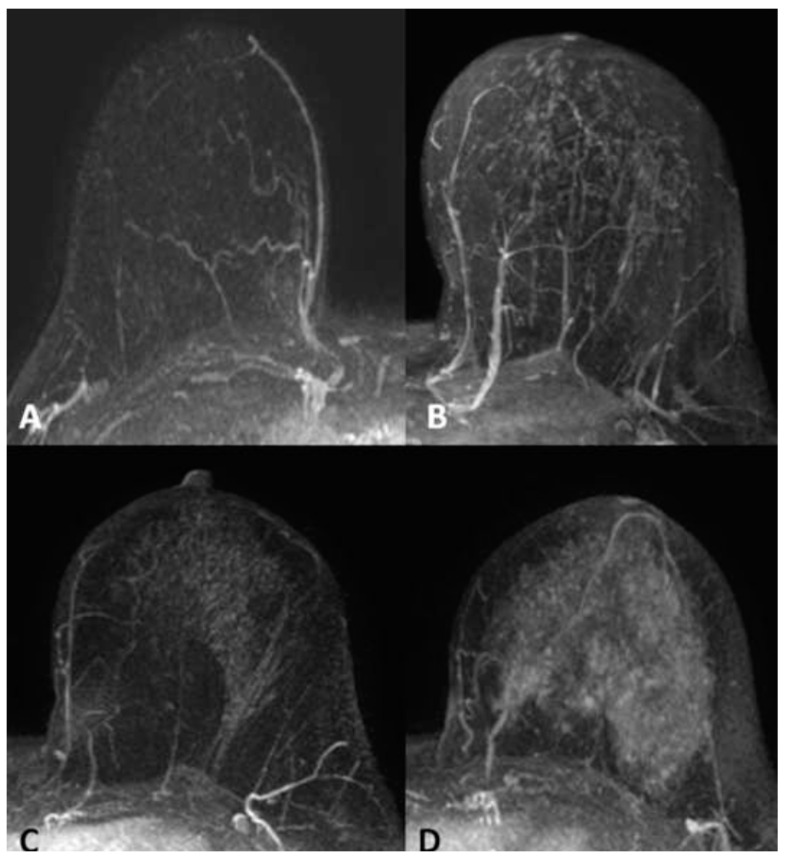
Three-dimensional maximum intensity projection (MIP) MR-enhanced images. BPE classification: (**A**) Type 1—Minimal. (**B**) Type 2—Mild. (**C**) Type 3—Moderate. (**D**) Type 4—Marked.

**Figure 2:**
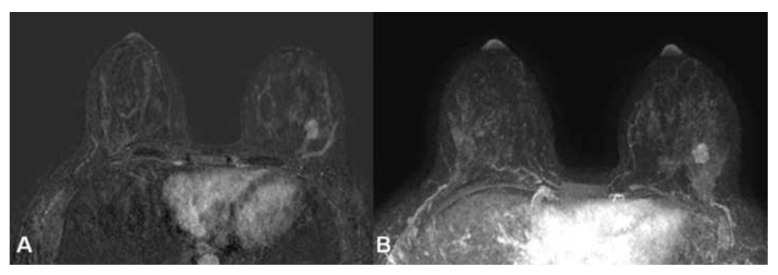
Subtracted THRIVE (**A**) and 3D MIP (**B**) contrast-enhanced MRI showing a NST breast cancer on the left side in a 44-year-old HL survivor with Type 3 BPE evaluated in the contralateral breast.

In the HL group, four cases of breast cancer were detected (Figure 1 and Figure 2), corresponding to a 6.5% incidence rate (four out of sixty-two women). In comparison, no cases of breast cancer were detected in the control group during the study period.

HL Survivors: in the group of HL survivors, BPE distribution (Table 1) was observed as follows:

- Minimal BPE (Type 1): 12.9% (8 women);
- Mild BPE (Type 2): 25.8% (16 women);
- Moderate BPE (Type 3): 41.9% (26 women);
- Marked BPE (Type 4): 19.4% (12 women).

Control Group: in the control group of high-risk women (BRCA mutation carriers), BPE distribution was observed as follows:

- Minimal BPE (Type 1): 19.4% (12 women);
- Mild BPE (Type 2): 35.5% (22 women);
- Moderate BPE (Type 3): 32.3% (20 women);
- Marked BPE (Type 4): 12.9% (8 women).

In HL survivors, BPE was significantly higher in the irradiated breast compared to the contralateral breast (*p* = 0.02). This suggests a potential correlation between previous radiation therapy and increased BPE in the treated breast tissue.

Further analysis revealed that BPE was more pronounced in patients who received radiotherapy at an earlier age, particularly those who had been treated before the age of 25 (*p* = 0.03).

A multivariate regression analysis (Table 2) was performed to assess the influence of variables such as age at treatment, time since radiotherapy, and BPE on breast cancer risk. The results indicated that increased BPE was associated with a higher risk of developing breast cancer in both groups. Specifically, a marked BPE (Type 4) was significantly correlated with a higher incidence of breast cancer (*p* = 0.01).

The inter-rater agreement between the two radiologists for BPE classification was assessed using Cohen’s kappa statistic. The agreement was found to be 0.82, indicating almost perfect agreement. In cases of disagreement, a third radiologist was consulted, and a consensus was reached in all instances.

Results of comparison of BPE between HL survivors and control group was as follows: Statistical analysis using the χ^2^ test revealed a significant difference in BPE distribution between HL survivors and control subjects. HL survivors demonstrated a higher prevalence of moderate (Type 3) and marked (Type 4) BPE compared to the control group (*p* = 0.04).

Among the HL survivors, the detection of malignant lesions was found to correlate with marked BPE (Type 4). Out of the four malignant lesions identified, 75% (three out of four) occurred in women with Type 4 BPE. No malignant lesions were observed in the control group.

## 4. Discussion

In recent decades, the outcomes of treatment for early-stage Hodgkin lymphoma (HL) have significantly improved. However, as survival rates have increased, the challenges of life after treatment have become more pressing, with long-term effects now emerging as a greater concern than the treatment itself. Common late effects include secondary cancers, cardiovascular diseases, thyroid dysfunction, early menopause, and fatigue. These sequelae can negatively impact the health of survivors and contribute to premature mortality [1,7,8,21,22].

In the first ten years following diagnosis, mortality is mainly attributable to HL itself. However, with prolonged follow-up, treatment-related complications, particularly secondary cancers and cardiovascular diseases, emerge as the leading causes of excess mortality. These complications also show sex-specific trends in this relatively young cohort [1,2,4].

Among long-term survivors, the most common secondary cancers are breast cancer (BC) in women and lung cancer in men. Notably, women treated with radiotherapy (RT) for HL are at significantly higher risk of developing BC compared to the general population [3,5,23,24,25].

Despite the development of numerous new targeted agents for the treatment of HL, the role of RT remains debated. Current RT strategies focus on reducing irradiated fields to minimize late effects. It is well established that involved-field radiation therapy (IF-RT) is as effective as extended-field RT but with less toxicity. As a result, research interest in RT primarily centers on reducing the size of irradiated fields [26]. In our study, all patients were treated with IF-RT, consistent with current practices to reduce the risk of late effects.

Some studies suggest that ABVD chemotherapy alone might suffice for patients with stage IA or IIA HL, but this strategy remains controversial and has not been widely confirmed by large clinical trials. This has stimulated the development of new, less harmful RT techniques. One such advance is involved-node RT (IN-RT), which targets enlarged lymph nodes after chemotherapy rather than the entire nodal region [27,28]. Although future randomized trials are needed to establish whether IN-RT should become the standard treatment, it represents a promising approach.

Recent advances, such as helical tomotherapy (TOMO), have shown potential in reducing radiation doses to critical organs, including the breast, lungs, heart, and thyroid, in HL patients [29]. TOMO allows treatments that are not possible with conventional RT methods, such as simultaneous irradiation of multiple mediastinal lymph nodes. While no randomized trial directly compares different RT approaches, ILROG guidelines recommend using involved-site radiation therapy (ISRT) over IF-RT in the early phase after ABVD chemotherapy [30].

A large, randomized study, the H10 study, did not demonstrate the non-inferiority of ABVD alone compared to combined treatment in patients with a negative interim PET scan (Deauville score two) [31]. However, because patients treated with ABVD alone often have a good overall prognosis, this approach might be considered for individual patients when long-term RT risks outweigh the short-term benefits of better disease control.

Specialized follow-up and breast cancer screening are recommended for women at high risk of secondary BC to ensure early detection and optimal management [13,14,32]. Consensus guidelines suggest annual breast cancer surveillance with mammography, magnetic resonance imaging (MRI), or a combination of both for women exposed to chest radiation, starting at least 8 years after exposure [4,13,14,20,23,33].

The age at which treatment for HL is administered appears to be one of the most significant risk factors for developing secondary BC. Women treated during puberty may be particularly at risk, likely due to the greater sensitivity of breast tissue to radiation during this period of rapid cell proliferation [4,24].

Secondary breast cancer in HL survivors is often diagnosed at least 20 years earlier than in the general population, with a median age of diagnosis around 40 years. These tumors are frequently bilateral, located at the edges of the RT field, and tend to occur in the outer quadrants of the breast [4,5,6,23,24,25,34]. In our study, three cases of breast cancer were detected 15 years after treatment, all in women with a history of chest radiation therapy.

A link between background parenchymal enhancement (BPE) and breast cancer risk has been proposed, with BPE potentially serving as a biomarker for breast cancer risk. However, MRI sensitivity decreases significantly in women with moderate and marked BPE compared to those with minimal or mild BPE, due to reduced contrast resolution in enhanced MRI [11,12,19]. Zeng et al. reported that BPE in breast MRI screening for breast cancer in patients treated with RT for HL is significantly higher compared to matched controls. They observed a positive correlation between BPE and age in HL survivors, but a negative correlation in the control group, suggesting that the increase in BPE in HL survivors may reflect an elevated breast cancer risk due to long-term radiation damage to the breast microvasculature and chronic inflammation [35].

In our study, we found that the BPE patterns in HL survivors were comparable to those in high-risk women, such as BRCA mutation carriers. Specifically, 31 HL survivors (50%) showed moderate or marked BPE, similar to the 30 (48%) cases in the BRCA mutation carrier control group. In the HL survivor group, three malignant tumors were detected: two cases of ductal carcinoma in situ (DCIS) and one of invasive carcinoma NST. Notably, two of these cases (DCIS and NST) had lesions with moderate BPE in the contralateral breast, while the third case (NST) showed marked BPE.

The fact that BPE patterns in HL survivors closely resemble those in high-risk women suggests that long-term radiation therapy may induce a breast tissue profile similar to that seen in genetically predisposed women. This highlights the potential of BPE as a useful imaging biomarker to assess breast cancer risk in HL survivors, who may face similar long-term risks as genetically predisposed women.

Although MRI is generally recommended for women at high risk of breast cancer, some studies have explored its potential in women at moderate risk. However, these studies have produced conflicting results, with one study finding a positive correlation between BPE and breast cancer, while another found no such association. A limitation of these studies is that MRI was performed in women with symptoms, such as bloody nipple discharge, or in women already diagnosed with cancer. Therefore, the applicability of these results to asymptomatic women remains unclear [11,12,19,36].

A more comprehensive analysis of breast cancer risk, particularly with the advent of new targeted therapies (e.g., nivolumab and brentuximab Vedotin), may help refine therapeutic decisions and reduce the risk of secondary cancers [37,38].

In our study, patients received a relatively low chemotherapy load, with only four cycles of the ABVD regimen, which is known to be less intensive compared to other chemotherapy regimens. However, even with a limited chemotherapy load, chemotherapy can still negatively influence the development of secondary cancers, including breast cancer. The drugs in the ABVD regimen (adriamycin, bleomycin, vinblastine, and dacarbazine) are known for their DNA and immune system toxicity, which may predispose patients to a higher risk of developing long-term malignancies. Although the reduced number of cycles decreases the likelihood of severe side effects compared to more intensive treatments, exposure to a reduced chemotherapy load can still contribute to secondary cancers, especially when combined with radiotherapy, which locally increases tissue sensitivity to oncogene mutations. Therefore, it is challenging to fully distinguish the specific effects of chemotherapy from those of radiotherapy, as both therapies have the potential to alter tissues and DNA, promoting the development of long-term tumors.

### Limitations of the Study

Young age of participants: the study primarily focused on a relatively young cohort, which may not fully represent the older HL survivor population, who may experience different long-term effects.

Follow-up duration: the median time from radiotherapy was 15 years. However, secondary cancers typically emerge after 20 years, suggesting that a longer follow-up period would be necessary to better understand long-term cancer risk.

Comparison with non-BRCA mutation carriers: The control group consisted of women with BRCA1 or BRCA2 mutations but not those without such genetic mutations. Including a group of women without genetic mutations could provide further insights into the differences in breast cancer risk.

Monotherapy: the study exclusively evaluated patients treated with involved-field radiation therapy and did not consider other RT techniques or chemotherapy regimens, which might have different impacts on the risk of secondary cancer.

Sample size: the relatively small sample size (62 women per group) limits the generalizability of the results, and larger studies are needed to confirm these findings.

The innovative aspect of this study lies in its focus on assessing breast cancer risk in HL survivors using background parenchymal enhancement (BPE) on MRI, particularly in a homogeneous cohort of patients with well-defined diagnoses and uniform treatment with chemotherapy and radiotherapy. Although it is well-known that radiotherapy (RT) increases the risk of secondary breast cancer in HL survivors, few studies have specifically explored the role of MRI-based biomarkers, such as BPE, in predicting this risk. Our study is distinctive because it directly compares BPE patterns in HL survivors treated with involved-field radiation therapy (IF-RT) with those of high-risk women, including BRCA mutation carriers, who are genetically predisposed to breast cancer. By focusing on a homogeneous group of HL survivors, all treated with a similar regimen of four cycles of ABVD chemotherapy and IF-RT, we minimize confounding factors and offer a clearer assessment of how these specific treatments influence BPE and, consequently, breast cancer risk.

## 5. Conclusions

The incidence of breast cancer found in this study was relatively low (5%) among Hodgkin lymphoma survivors treated with involved-field radiation therapy. The use of more targeted radiation techniques, such as involved-node radiation (IN-RT), along with the introduction of first-line targeted therapies, may further reduce the risk of secondary tumors. Future research should focus on exploring the underlying cancer biology and genetic susceptibility to treatment-induced tumors to better tailor therapies and minimize long-term risks.

## Figures and Tables

**Table 1:** Distribution of the BPE types in HL survivors and age-matched control group.

	BPE1	BPE2	BPE3	BPE4	* p * Value
HL SURVIVORS	13 (21%)	18 (29%)	16 (26%)	15 (24%)	* p * = 0.98
CONTROL	12 (19%)	20 (32%)	15 (24%)	15 (24%)	

**Table 2:** Multivariate regression analysis on BPE distribution in HL survivors.

	Mean	Standard Deviation	R	* p *	Multivariate Regression
Age	35.56	7.56	−0.07	0.22	R2 = 0.08 *p* = 0.17
Age at treatment	19.16	7.65	0.1	0.11	
Years from treatment	15.88	6.66	0.06	0.35	

R: coefficient of determination for the single independent variables. R2: coefficient of determination for the multivariate regression analysis.

## Data Availability

The relevant data have been included in the manuscript. The datasets used and/or analyzed during the current study are available from the corresponding author upon reasonable request.

## Abbreviations

HL	Hodgkin Lymphoma
RT	Radiotherapy
IF-RT	Involved Field Radiotherapy
ABVD	Adriamycin, Bleomycin, Vinblastine, Dacarbazine (chemotherapy regimen)
BPE	Background Parenchymal Enhancement
BC	Breast Cancer
MRI	Magnetic Resonance Imaging
DBT	Digital Breast Tomosynthesis
3DCRT	3D Conformal Radiotherapy
DCIS	Ductal Carcinoma In Situ
NST	Non-Special Type (of breast cancer)
MIP	Maximum Intensity Projection
T1WI	T1-Weighted Imaging (MRI sequence)
T2WI	T2-Weighted Imaging (MRI sequence)
STIR	Short TI Inversion Recovery (MRI sequence)
THRIVE	T1 High Resolution Isotropic Volume (MRI sequence)
FOV	Field of View
TR	Repetition Time (MRI parameter)
TE	Echo Time (MRI parameter)
TI	Inversion Time (MRI parameter)
SENSE	Sensitivity Encoding (MRI technique)
χ^2^	Chi-Square Test (statistical test)
κ	Cohen’s Kappa (inter-rater agreement statistic)
